# Biodegradable Films Prepared from Pulp Lignocellulose Adhesives of Urea Formaldehyde Resin Modified by Biosulfonate

**DOI:** 10.3390/polym14142863

**Published:** 2022-07-14

**Authors:** Yongjie Ma, Yanxin Luo, Qiannan Zhang, Yanming Gao, Jianshe Li, Sadiq Shah, Xiaozhuo Wang, Xueyan Zhang

**Affiliations:** 1 School of Agriculture, Ningxia University, Yinchuan 750021, China; mayongjie0526@163.com (Y.M.); lyx2574245547@163.com (Y.L.); zqn2949525151@163.com (Q.Z.); myangao@163.com (Y.G.); jslinxcn@163.com (J.L.); 2 Abdul Wali Khan Univ Dept Food Sci & Technol, Garden Campus, Mardan 23200, Pakistan; sadiqshah@awkum.edu.pk

**Keywords:** bio-based sulfonate, lignin, modified urea-formaldehyde resin, biodegradable film

## Abstract

Traditional low-density polyethylene (LDPE) film causes environmental pollution; there is a pressing need to make new bio-based polymers for alternative products, to meet agricultural production needs and for sustainable ecological development. In this study, urea-formaldehyde resin (UF) was modified with polyvinyl alcohol (PVA) and 1–2.5% bio-based sulfonate (BBS). The influence of BBS inducing on the functional groups, microstructure, and thermal behavior was evaluated by Fourier transform infrared spectroscopy (FTIR), scanning electron microscopy (SEM), differential scanning calorimetry (DSC), and thermogravimetric analysis (TGA). A biodegradable film was prepared with modified UF resin as adhesive and pulp lignocellulose as raw material. The biodegradable mulch film samples were tested for biodegradability, water retention, and cooling soil temperature characters using LDPE and no mulching (NM) as a control. The results showed that with the increase of BBS content, the viscosity and reactivity of modified PUF resin increased, and the free formaldehyde content decreased. A 2%BBS modified PUF resin (2.0BBS/PUF) accelerated the curing process of the PUF resin, formed a flexible macromolecular network structure, and enhanced the toughness of the resin. The biodegradable mulch prepared with PUF, BBS, and 2.0BBS/PUF as adhesives had good water retention. BBS modification increased the degradation rate of mulch by 17.53% compared to the PUF. Three biodegradable films compared with LDPE and NM significantly reduced the soil temperature under summer cucumber cultivation, and the 2.0BBS/PUF coating had the lowest diurnal temperature difference, which provided a suitable soil environment for crop growth.

## 1. Introduction

Plastic mulching has been proven to be beneficial for agricultural production in conserving water [1], increasing surface soil temperature, modifying microclimates [2], and inhibiting weed growth [3]. Moreover, plastic mulching creates suitable conditions for plant development and high fruit yield and quality, and reduces the requirements for water, herbicide, and fertilizer, which contributes to meeting the growing demand for food to sustain the escalating population [4,5]. China introduced mulch coating technology from Japan in the 1970s and is currently the country with most widespread use of this method in the world [6]. LDPE has been widely used due to its versatile nature and effectiveness. However, LDPE cannot be degraded in the short term after use [7]. The long-term use of LDPE mulch film causes residues to accumulate in the soil, destroying the soil structure and hindering the exchange of water, heat, and air between the soil and air. This leads to a decline in cultivated land quality and restricts the healthy production of vegetable crops [8].

To address the problem of environmental pollution caused by the extensive use of LDPE mulch film, a new biodegradable film made from natural polymers, including starch, cellulose, and lignin, is considered an adequate substitute [9,10,11]. Compared with LDPE film, biodegradable film is decomposed into CO_2_, H_2_O, CH_4_, inorganic compounds, and soil nutrients into the soil after use. These materials have no adverse effects on the environment, crops, or humans [12]. In recent years, incorporating natural biopolymers into biodegradable materials has been extensively studied, providing new or improved ideas for developing biodegradable films [13,14]. The common technique is to incorporate biopolymers as reinforcements, directly or by spraying, into the film-forming solution, or by casting, extruding, or blowing the film [15,16]. Pulp is rich in cellulose and hemicellulose, which can be used as an ideal material for biodegradable films [17]. As the world’s primary pulp and paperboard producer, China produces many harmless solid wastes every year. Xuan paper waste is the solid waste produced during production, and currently, about 1000 tons are produced every year in China, and over 2000 tons of xuan paper waste are created, occupying considerable land and resulting in a wasted resource [18]. The industry has been actively exploring new methods of waste resource utilization [19]. Previous studies reported the effect of polyethylene compatibilizing agents on the mechanical and thermal properties of waste white paper-filled low-density polyethylene composites. They found that adding a compatibilizing agent can enhance the filler dispersion in the matrix and improve the mechanical properties [20]. This provides innovative ideas for the research and development of biodegradable mulch films. The biodegradable film’s raw material has to meet practicability and cost considerations, so it is required to find a more suitable solubilizer. 

UF is a polymer formed by the polycondensation of urea and formaldehyde, one of the essential members of thermosetting resins for bonding substrates [21,22]. UF resin has a high reactivity, adhesion, and water solubility properties, and also has a low curing temperatures, short press time, is easy to prepare, and has a relatively low cost [23,24,25]. UF can be considered as an adhesive for biodegradable films. However, the formaldehyde-urea reaction is reversible. Urea-formaldehyde resin synthesized by the traditional process has a high formaldehyde content and poor mechanical properties, which need to be modified [26,27]. The most common methods to reduce the formaldehyde emission from UF is by modifying the adhesive or using formaldehyde scavengers, especially reducing the molar ratio of formaldehyde and urea in UF resin [28]. Introducing highly active compounds such as melamine or polyvinyl alcohol into a UF resin can enhance its properties and reduce the formaldehyde content [22,29]. Polyvinyl alcohol (PVA) is a synthetic thermoplastic hydrophilic polymer with transparency and good mechanical and biodegradability [30]. PVA has great potential for improving UF performance [31]; in a previous study, PVA was effectively used to enhance the toughness of UF resin and reduce formaldehyde content [32]. This is mainly because PVA contains many hydroxyl groups, which react with formaldehyde to form polyvinyl formal under acidic conditions, the hydrophilic hydroxymethyl content of UF is reduced, and the adhesive’s water resistance and initial viscosity are improved [33]. In addition, the structure formed by the reaction of PVA with formaldehyde increases the branching design and crosslinking ability of the cured resin, thus capturing the residual formaldehyde in the adhesive [34]. However, the improvement of PVA for the storage stability and crystallization properties of UF is very limited [34]; as a binder for preparing biodegradable film, this performance needs to be improved.

Lignin is generally underutilized as a by-product in the biorefinery industry, is a polyaromatic amorphous macromolecule, and is the second most abundant natural raw material, followed by cellulose [35]. As lignin contains phenolic hydroxyl, aliphatic hydroxyl, and carboxyl functional groups, it has been widely used as a modifier in the synthesis of various sustainable development materials [36]. The polyphenol structure of lignin is the main reason for its application in binder composition. Lignin is considered an adequate substitute for petroleum-based phenol, to replace phenol in phenol-formaldehyde adhesives and improve the performance of formaldehyde-based synthetic resins [37]. Lignosulfonate contains many aliphatic hydroxyl and sulfonate groups with an excellent surface activity and is proven to be suitable for modification or as a binder, dispersant, or reinforcing material in polymers [38]. Lignosulfonates have high reactivity with formaldehyde and have been used as formaldehyde scavengers in the case of particleboards, fiberboards, plywood, etc. [39,40]. In addition to active sites, the number of aliphatic and phenolic hydroxyl groups increases the reactivity of lignin to synthetic adhesives such as aldehydes, tannins, and phenols [41]. Therefore, it is promising to use lignosulfonate as a filler in UF resin, to effectively improve the reactivity of the polymer, which may be industrialized in the production of UF resin [42].

This study employed bio-based sulfonate (BBS), derived from the sulfonation system SO3/DCE, to reduce the formaldehyde content of polyvinyl alcohol urea-formaldehyde (PUF) resin, and used residual softwood fibers from the pulp and paper industry to produce a biodegradable film. This provides a new research concept for the resource utilization of papermaking waste and the development of a biodegradable film.

## 2. Materials and Methods

### 2.1. Materials

Formaldehyde (37 wt% water solution), polyvinyl alcohol-124, sodium hydroxide, ammonium chloride, acetic acid, and sodium carbonate were purchased from the Xilong Science Company (Shantou, China). Urea was provided by Guangdong Guanghua Technology Co., Ltd. (Guangdong, China). Sodium sulfite, boric acid, iodine, and potassium iodide were purchased from the Tianjin Combo Chemical Reagent Company (Tianjin, China). Bio-based sulfonate raw material was the product of agricultural straw waste. The high molecular weight and degree of sulfonation of the native pulp cellulose sulfate and transverse lignin salt was synthesized using an SO_3_/DCE (1,2-dichloroethane) sulfonation system. The main components were a mixture of cellulose, hemicellulose sulfate monoester, lignin sulfonate, and humic acid sulfonate. They were provided by Beijing Ziguang Yingli Chemical Technology Co., Ltd. (Beijing, China). The specific ingredients are shown in Table 1. A 4500A multifunctional pulverizer processed the paper powder with a speed of 32000 rpm and a pulverization degree of 250–350 mesh.

### 2.2. Synthesis and Preperation

#### 2.2.1. Synthesis of BBS-Modified PUF Resin

The modified urea-formaldehyde resin was prepared by the “alkali–acid–alkali” method in a four-necked flask fitted with a mechanical stirrer and a reflux condenser. The total molar ratio of urea to formaldehyde was 1.4 (U:F = 1:1.4). First, 37% formaldehyde solution was added to a three-necked flask, and the polymer solutions were heated to 40 °C under stirring. The modified urea-formaldehyde resin was adjusted to a pH of 7.5–8.0, and the first batch of urea U1 (F/U1 molar ratio is 2.0) was added with reaction for 10 min. Afterward, the material was heated to 60–65 °C, 4% (total mass relative to urea) polyvinyl alcohol-124 was added, and the heating continued to 90 °C for 60 min. Then, the pH value was adjusted to 4.8–5.1, the reaction was continued, the reaction endpoint was continuously determined, and the pH value was adjusted to 7.5–8.0, accompanied by the reaction endpoint. The sulfonates and second batch of urea U2 (F/(U1 + U2) mol ratio was 1.5) were added and reacted at 80–85 °C for 30 min. The third batch of urea U3 (F/(U1 + U2 + U3) mol ratio is 1.3) was added, reacted at 70–80 °C for 30 min, and the material pH value was adjusted to 7.5–8.0, then it was cooled to 40–50 °C and poured. The sulfonates quantities were determined as 0%, 1%, 1.5%, 2%, and 2.5% of the total mass of urea. They were expressed as PUF, 1.0BBS/PUF, 1.5BBS/PUF, 2.0BBS/PUF, and 2.5BBS/PUF, respectively.

#### 2.2.2. Preparation of Degradation Film

Biodegradable films were prepared using bio-based sulfonates and different amounts of bio-based sulfonate-modified urea-formaldehyde resins as adhesive. The mass ratio of paper powder, water, and adhesive was set as 1:30:2. The biodegradable film preparation process included three steps. First, add the mixed hybrid solution into the multifunctional reactor, heat and stir at 60 °C for 30 min, and then pour the mixed solution into a culture dish with sand at the bottom; the plate was placed in a drying oven at 30 °C for 48 h. Finally, obtain the cured biodegradable mulch sample.

### 2.3. Measurement

#### 2.3.1. Properties of Modification UF Resins

According to the national standard GB/T14074–2006 [43]. A PHSJ-3F (Leimagnetic Precision Instrument Co., Ltd, Shanghai, China.) audiometer with automatic temperature compensation was used to measure the pH value of UF resins. An NDJ-5S digital viscometer (Ander Instrument Co., Ltd, Shanghai, China.) measured the UF resin viscosities with a 27 °C ± 0.5 °C temperature and 60 rpm spinning rate. The gel time of the U.F. resin with different hardeners was measured using a boiling water bath. Each resin agent was parallelly measured at least three times.

The solid content (SC) was determined by the weighing method. The container was weighed, denoted as *m*1, and a 1 g sample was considered with the weighed container, designated as *m*2. The piece was placed in a constant-temperature oven and dried at 120 °C for 2 h. After removing the container, it was cooled in a dryer for 15 min, immediately weighed after removal, and denoted as *m*3. Each resin agent was parallelly measured at least three times, and SC was calculated as follows:

(1) $$\mathrm{SC}=\frac{m3-m1}{m2-m1}\times 100\%$$

where SC expresses solid content (%), *m*1 expresses the weight of the container (g), and *m*2 is the mass of the container and sample before drying (g). *m*3 is the mass of the container and sample after drying (g).

#### 2.3.2. Free Formaldehyde Content Measurement

The free formaldehyde content emission of UF was determined using the titration method and the procedure described in China National Standard GB/T14074-2006. Weigh 1.5 g of the sample and quickly dissolve it in a mixed solution consisting of 10 g of ice, 25 mL of boric acid buffer solution, and 150 mL of ice–water mixture; stir the mixture with a magnetic stirrer at 0 °C, and add 2 mL of 1 mol/L sodium sulfite solution during the process; continue to stir for 15 min, add 10 mL of 1mol/L acetic acid and 3–4 drops of 1% starch solution; titrate with iodine solution until gray–blue or purple appears and stabilize for at least 10 s. Add 30 mL of sodium carbonate solution, titrate the sodium sulfite released by the reaction with iodine solution until a blue color appears, and stabilize for at least 1 min, record the volume V of the iodine solution required to titrate the released sodium sulfite, and measure each sample twice and average the values. Use Formula (2) to calculate the amount of free formaldehyde released from the resin:

(2) $$w=\frac{V\times 1.5\times 0.1}{m}\times 100\%$$

where *w* expresses the free formaldehyde content, %; *V* expresses the volume of iodine solution consumed by the sodium sulfite released by the titration reaction, mL; *m* expresses the sample mass, g; 1.5 is the equivalent of the abundance of free formaldehyde content in 1.00 mL c(1/2 I_2_) = 0.1 mol/L iodine solution; 0.1 expresses the factor to convert mg to g and *w* to percent.

### 2.4. Sample Characterization Analysis

After freezing for 12 h in a DW-86L626 ultra-low temperature storage box (Haier Special Electric Appliance Co., Ltd, Qingdao, China.), each processed sample was placed in an Alpha1-4/2-4LD Plus vacuum freeze dryer (Christ, Germany) for freeze-drying and grinding. Then, they were powdered and stored in dry Petri dishes for characterization testing.

#### 2.4.1. Fourier-Transform Infrared (FTIR) Spectroscopy

Molecular-level changes in the resin during modification were recorded using Fourier transform infrared spectroscopy (FT-IR) (Nicolet iS50-Thermo Scientific, Commonwealth of Massachusetts, Waltham, MA, USA) with 32 scans over a wavenumber range of 4000–500 cm^−1^ at a 4 cm^−1^ resolution.

#### 2.4.2. Thermal Stability of Resins

A thermogravimetry thermal analyzer (EXSTAR series TG7200, SII Nano Technology Inc., Tokyo, Japan.) was used to record the thermal properties of the resin structure, with samples heated from 30 °C to 600 °C at a heating rate of 10 °C/min under an N_2_ atmosphere.

#### 2.4.3. Differential Scanning Calorimetry (DSC) Analysis

First, 6.5 mg of the freeze-dried sample was placed in an aluminum pan. Then, the pan was sealed and heated from 25 to 250 °C at a heating rate of 20 °C/min using a TA Instrument (DSC Q20, Waters Company, New Castle, DE, USA). Each measurement was performed under an N_2_ flow with a 50 mL/min flow rate.

#### 2.4.4. Scanning Electron Microscope (SEM) Analysis

The sample was sputter-coated for 10 min with gold using an ion sputter (Hitachi E-1010 Ion Sputter, Tokyo, Japan), and then a Hitachi S-3400N (Hitachi Science System, Ibaraki, Tokyo, Japan) scanning electron microscope was used to observe the resultant adhesives.

### 2.5. Degradable Film Performance

#### 2.5.1. Water Retention

Potting soil was passed through a 1-mm sieve and dried to a constant weight at 105 °C. A Petri dish with a diameter of 9 cm contained 80 g of dried soil, 30 g of water was sprayed, and then the same diameter size of biodegradable mulching was immediately placed on the top. The weight was measured and recorded as *m*_0_. The dishes were then cultivated in a climate-controlled chamber for 24 h, at 60% air relative humidity, temperature 28 °C, and with 16 h light per day. The dish weight was measured and recorded as mi every two hours. The percentage of mass loss was used to represent the water retention of mulch film, and the formula below was followed:

(3) $$m=\left(1-\frac{m_{0}-m_{i}}{30}\right)\times 100\%$$

where *m* expresses the mass loss rate, *m*_0_ is the original weight, and *m_i_* is the weight after *i* hours (*i* = 2, 4, 6…).

#### 2.5.2. Soil Temperature under Films

Soil temperature recorders (QM-ZC-16, Shangqiu Sensor Technology Co. Ltd., Henan, China) were installed at a 15 cm soil depth in the middle row of various plots. The soil temperature was monitored every 30 min and recorded on a memory card. The mean daily soil temperature was calculated based on the measured data.

#### 2.5.3. Degradation Rate of Mulch Film

The samples with different treatments were buried in the soil at a depth of 10 cm, with a humidity of 60%, taken out after 120 days, and the remaining mulch fragments were collected, washed, dried, and weighed [44]. Weight loss (*M*) was calculated from the weight change before (*M*0) and after biodegradation (*M*1):

(4) $$W=\frac{\left(M0-M1\right)}{M0}\times 100\%$$

where W expresses the degradation rate, *M*0 expresses the original weight before being buried in the ground, and *M*1 expresses the weight after being buried in the ground for a certain period.

### 2.6. Statistical Analysis

Statistical data were analyzed with Excel 2016 (Microsoft, WSU, Redmond, WA, USA) and SPSS 24.0 (International Business Machines Corporation, NY, USA). One-way ANOVA analyzed the mean values, with a *p* < 0.05. Chart drawing was performed using Origin 2021 and PowerPoint 2016 (Microsoft, WSU, USA) software.

## 3. Results

### 3.1. Physical Properties of Resin

**Figure 1:**
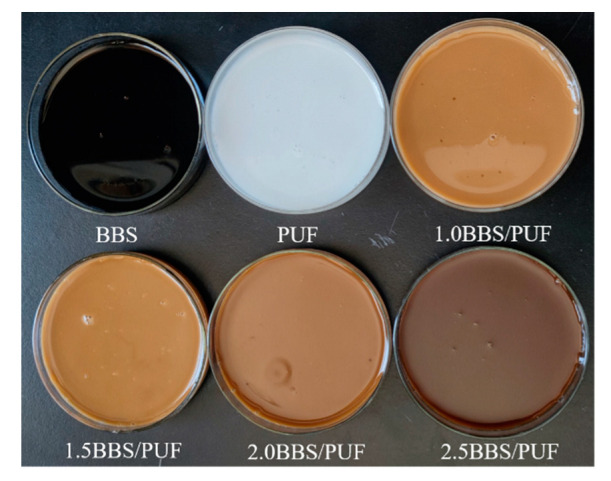
The appearance of BBS, PUF, and BBS modified PUF resins with different additions. BBS: bio-based sulfonate; PUF: Polyvinyl alcohol modified urea-formaldehyde resin; 1.0BBS/PUF: 1% BBS modified PUF resin; 1.5BBS/PUF: 1.5% BBS modified PUF resin; 2.0BBS/PUF: 2% BBS modified PUF resin; 2.5BBS/PUF: 2.5% BBS modified PUF resin.

Figure 1 shows the physical appearance of the BBS, PUF, and BBS-modified PUF resin samples. Bio-based sulfonates (cellulose sulfate, lignosulfonate, straw sulfate, etc.) have good biocompatibility, and they can be used as a green binder to replace PUF resin or as a film-forming material for a slow-release fertilizer [45]. The pure PUF resin was white colloidal, and the pure BBS was a black solution. It was a kind of biological macromolecule material. With an increase of BBS addition, the color of the modified resin gradually deepened, which might have been the result of a condensation reaction between the hydroxyl group in the molecular structure of BBS and the formaldehyde in the PUF resin.

Table 2 shows the physical properties of the PUF resins modified with BBS addition. Compared with PUF, the pH, viscosity, solid content, and curing time of the BBS-modified PUF resins increased, while the free formaldehyde content decreased. The introduction of BBS increased the molecular weight of modified PUF, increasing the solid content. The curing time reflected the resin reactivity. The curing time increased with the increase of BBS content, mainly because the number of molecular branches in the polymer was increased by BBS modification, which improved the reactivity of the PUF resin [22]. Viscosity and free formaldehyde content are important evaluation indexes of UF-modified resins in practical applications; when the content of BBS increased from 0 to 2.5%, the viscosity of modified resin increased sharply from 297.97 mPa.s to 4043.20 mPa.s, and the content of free formaldehyde content decreased from 0.54 % to 0.26%. On the one hand, BBS is a kind of adhesive with good viscosity. On the other hand, the increase of BBS content significantly improves the interfacial compatibility of composites and increases the viscosity. BBS is also a surfactant, and its molecular structure contains many phenolic hydroxyl groups and aromatic protons of the guaiacyl units, sulfonic acid group, aliphatic side chains, etc. The existence of these groups improved the hydroxymethylation reaction of BBS to formaldehyde [46]. Therefore, with increased BBS content, the activation effect gradually decreased the free formaldehyde content [47,48]. The results of this experiment are consistent with previous studies, where using lignosulfonates as additives for wood composites resulted in a reduced free formaldehyde content [49,50].

### 3.2. Characterization Analysis of BBS and PUF Resin

**Figure 2:**
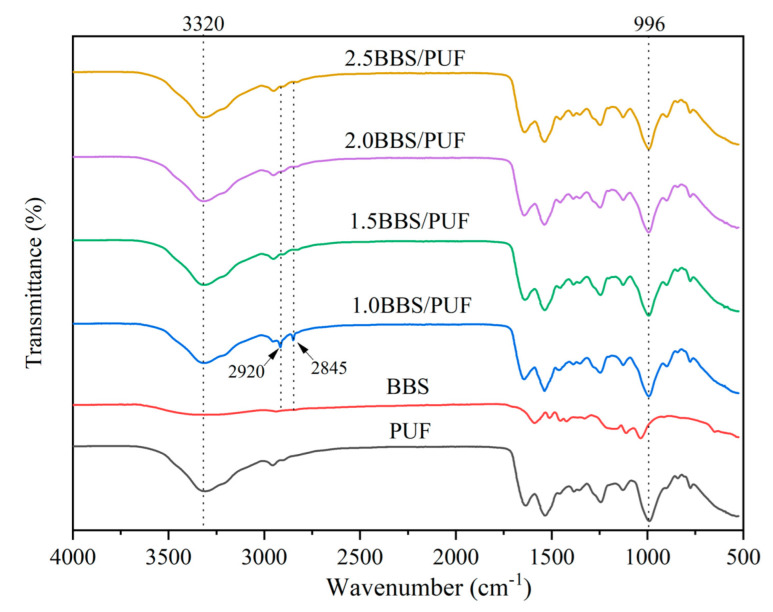
FTIR spectra of BBS, PUF, and BBS-modified PUF resins in the region of 500–4000 cm^−1^.

Figure 2 shows the FTIR absorption spectra of BBS, PUF, and BBS-modified PUF resins. The characteristic absorption peak of BBS at 3350 cm^−1^ belongs to aromatic and aliphatic O-H groups, and the ammonium group mainly causes the ammonium lignosulfonate in BBS. In addition, due to the mutual reaction between urea-formaldehyde and cellulose, hydrogen is formed, causing in the peak to shift to a lower wave number (3320 cm^−1^). The vibrations at 1600 cm^−1^, 1515 cm^−1^, and 1425 cm^−1^ correspond to the aromatic absorption peaks of the phenylpropane 9C skeleton [51]. There were strong absorption peaks at 1112 cm^−1^ and 1033 cm^−1^, mainly aromatic C-H, C-O, and C=O groups. It can be seen from the absorption peak intensity at 3320 cm^−1^ and 1367 cm^−1^ that there are a large number of aliphatic and phenolic hydroxyl groups in BBS, which is beneficial for the reaction of BBS with UF resin as the modifier [52]. The spectra of PUF and BBS-modified PUF resins are very similar, but there are some differences in specific chemical bands. After hydroxymethylation, the absorption peak at 3320 cm^−1^ is more obvious, due to the formation of N-H and O-H groups in the amino group, which indicates a reaction between the benzene ring and formaldehyde group [53]. Typical characteristic peaks of C-H and O-H stretching vibrations of cellulose/hemicellulose and lignin were found at 2920 cm^−1^ and 2845 cm^−1^, respectively [54]. Compared with PUF, these two peaks in BBS-modified PUF resin were more obvious, indicating that the cellulose and lignin in BBS reacted with PUF resin, and the peak gradually weakened with the increase of BBS addition. This was mainly because the reaction activity increased with the increase of BBS addition, which was consistent with the change of curing time in Table 2. The peak at 996 cm^−1^ corresponds to C-O in dimethyl urea [55], indicating that the resin contains more branched-chain structures, which may be related to the crystallinity and microstructure of the resin, and which is conducive to the formation of more crystal area and spherical particles [56].

**Figure 3:**
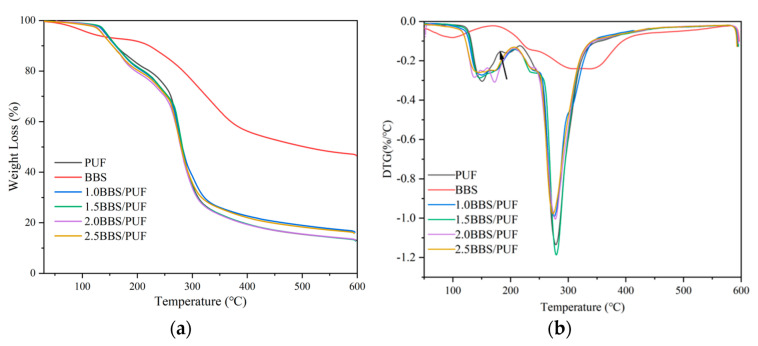
Thermogravimetric (**a**) and derivative thermogravimetric (**b**) curves of BBS, PUF resin, and BBS-modified PUF resins with different BBS additions.

To investigate the thermal stability of BBS and modified PUF resins, thermo-gravimetric (TG) and derivative thermogravimetry (DTG) analyses were carried out. It can be seen from Figure 3a that the thermal degradation of each sample is divided into four stages. The first stage is 30~125 °C, due to the evaporation of bound water in lignin (BBS) [57]. The intermolecular and intramolecular interactions in the modified resin form carbon and hydrogen bonds, resulting in a small amount of weight loss during resin curing [58]. The second stage is 125~250 °C; the mass loss was mainly due to the condensation reaction of amine and hydroxymethyl at high temperatures to release formaldehyde [59]. As shown in Figure 3b, the PUF resin showed an obvious peak at 150~200 °C, while the BBS-modified PUF resin did not appear, which showed that BBS-modified resin had an obvious inhibitory effect on the release of free formaldehyde [60]. The temperature range 250~350 °C was the primary thermal degradation stage of the wax, and the maximum pyrolysis temperature appeared at about 277 °C (Figure 3b). This stage mainly degraded the unstable chemical bonds and small molecular substances, such as the decomposition of methylene ether bridge into methylene bridge [61]. The fourth stage is the carbon formation stage at 350~600 °C. In this stage, the peptide bond was degraded into CO, CO_2_, and NH_3_ gas volatilization, resulting in weight loss [22]. The final residues of BBS, PUF, 1.0BBS/PUF, 1.0BBS/PUF, 1.5BBS/PUF, 2.0BBS/PUF, and 2.5BBS/PUF, were 46.55%, 12.96%, 16.22%, 12.79%, 13.04%, and 15.75%, respectively. Ammonium lignosulfonate residues mainly caused the residues of BBS [62]. Compared with PUF, 1.0BBS/PUF and 2.5BBS/PUF residuals increased, which may have been due to the shielding effect of unreacted BBS, and they do not necessarily represent an increase in thermal stability [63]. Adding 1.5% and 2.0% produced a lower residual amount, which was beneficial to the degradation of the biodegradable film prepared using modified UF resin as an adhesive. 

**Figure 4:**
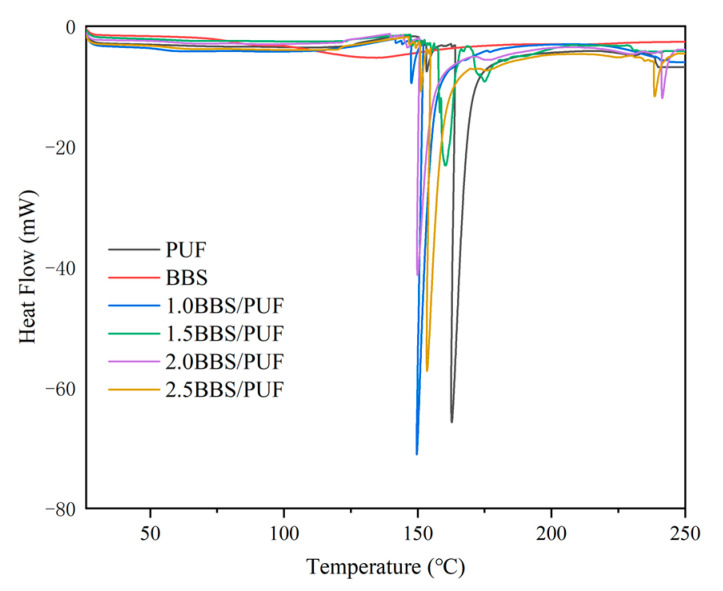
DSC curves of BBS, PUF resin, and BBS-modified PUF resins with different BBS additions.

The curing of the resin is crucial for bonding the substrate. As shown in Figure 4, the curing behavior of adhesive was analyzed using differential scanning calorimetry (DSC), the curing of modified PUF resin is an exothermic reaction, and there were apparent exothermic peaks in the different treatments. With the addition of BBS, the peak temperature of modified PUF resin decreased, and the temperature of 2% BBS modified PUF resin (2.0BBS/PUF) was the lowest, indicating that the modification of BBS accelerated the curing process of the PUF resin. This was mainly because the addition of BBS increased the content of hydroxymethyl and provided more reaction sites, to accelerate the curing of the resin system [64,65]. According to previous studies, the curing peaks with different lignin types in the resins changed between 130 °C and 150 °C, and the results of this experiment were consistent [66]. The curing peak temperature was slightly higher with partial addition, which may have been due to the increase of polymer viscosity due to the density of the BBS itself, and this reduced the diffusion and fluidity of UF resin molecules and thus reduced the reaction activity [67].

**Figure 5:**
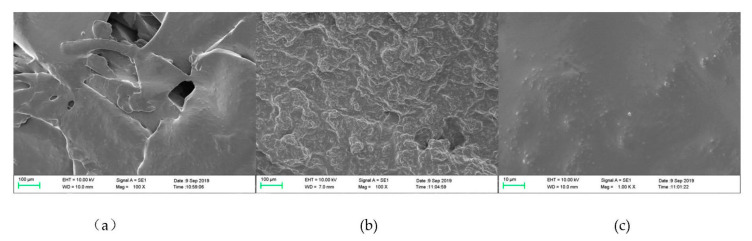
PUF (**a**), BBS modified PUF magnified 100 times (**b**), BBS modified PUF magnified 1000 times (**c**).

Figure 5a shows the SEM of PUF resin, and the inherent brittleness of UF resin produced a flat crystal structure and the appearance of holes on the surface in the PUF resin. This is because the hydrolysis reaction of PUF resin under high-temperature curing results in water and formaldehyde evaporation [56]. Figure 5b,c represents 100-fold and 1000-fold magnifications of 2.0 BBS/PUF, respectively. On the contrary, the surface of the 5b resin became rough and dense, indicating that the toughness of the resin increased. This was because of the reaction between the aldehyde groups in BBS and the hydroxyethyl urea in urea during the essential hydroxymethylation stage of resin synthesis, which participated in the acid condensation of the resin and situ polymerization with the PUF resin molecules. After curing, a flexible macromolecular network structure was formed, resulting in solid interaction and an enhanced toughness [22,29]. This shows that BBS modification improved the toughness of the PUF resin. A large number of spherical micro-particles appear in Figure 5c, which is considered to be related to the change of the resin crystals. The more complex crosslinking network structure of the UF resin was the main reason for the formation of spherical particles [68], which is consistent with the FTIR analysis results in Figure 2. 

### 3.3. Analysis of Film Characteristics

**Figure 6:**
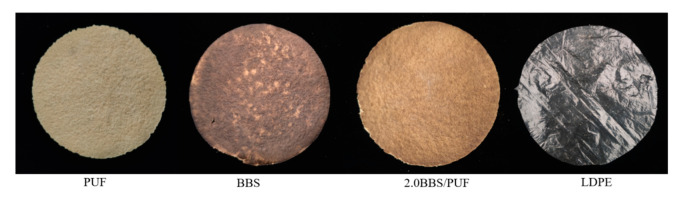
Differently treated mulch samples.

Different biodegradable film samples were prepared with PUF, BBS, and 2.0 BBS/PUF as adhesives (Figure 6). The color change of the film was the same as that of the cement. The practical application effect of the biodegradable film was explored by analyzing the degradation, water retention, and cooling of the biodegradable film.

**Figure 7:**
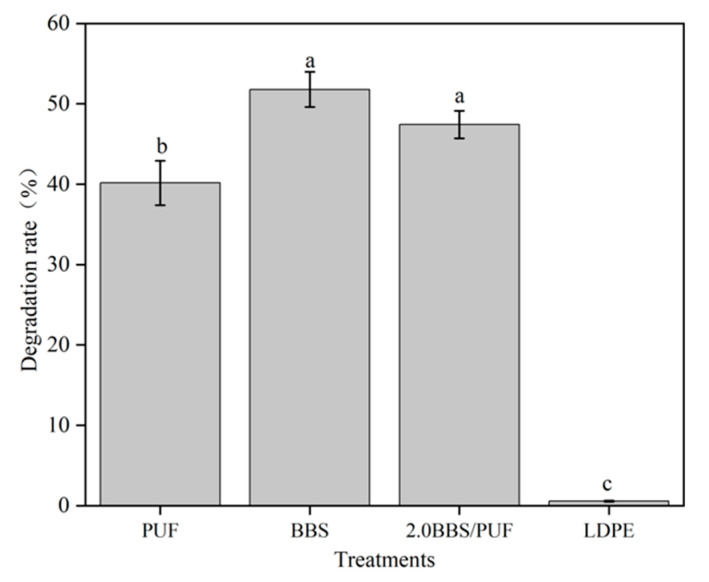
The degradation rate of different mulching films. Error bars represent standard deviations from five replicates. The lowercase letters in the bars indicate significant differences among the treatments (n = 6, *p* < 0.05).

Figure 7 shows the degradation rate of the different mulching films after 120 d of soil burial. The degradation rate of LDPE film was only 0.56% after 120 d, and the degradation rate of the three kinds of biodegradable film were all over 40%, while the degradation rates of BBS and 2% BBS/PUF were significantly higher than those of PUF film. This is consistent with the results of previous studies; the commonly used LDPE film does not degrade in a short time, while the biodegradable film degrades faster and the degradation rate is also different, which is related to the degradation time, soil microorganisms, and environmental conditions [69]. Compared with the PUF membrane, the degradation rate of the BBS-modified membrane increased by 17.53%, which may have been related to the structural changes of BBS itself. As shown in Figure 3, the thermal degradation peak of BBS appeared at a lower temperature (less than 100 °C) [70].

**Figure 8:**
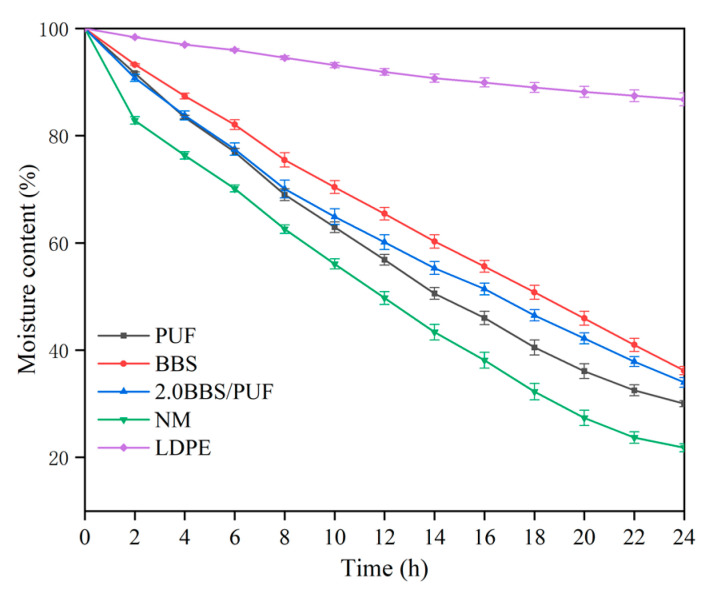
Water retention of different mulching films. Error bars represent standard deviations from five replicates.

As shown in Figure 8, the change of soil water content in 24 h under different plastic film mulching treatments indicated the water retention of the plastic film. With the extension of mulching time, the soil water content of each treatment gradually decreased. In the first two hours, the water content of PUF, BBS, 2.0 BBS/PUF, NM, and LDPE decreased by 8.36%, 6.76%, 9.24%, and 17.18%, and 1.63%, respectively. Compared with NM, the three biodegradable films effectively reduced soil water evaporation and maintained a relatively stable soil–water environment. After 24 h, the water content of each treatment decreased by 70.00%, 63.83%, 66.05%, 78.21%, and 13.26%, respectively. The LDPE film only had 13.26% water evaporation, which was mainly due to the condensation of a large number of water droplets under the LDPE film coating, which prevented the water and heat exchange between the soil and atmosphere, changed the water and heat cycle under the film, reduced soil evaporation, and effectively improved soil temperature and water content [71]. The BBS coating had the best water retention among the three biodegradable films, which may have been related to the composition of hydrophilic and hydrophobic compounds in the film. Previous studies showed that bioactive compounds with hydrophilic properties often increased the water vapor permeability of films [72]. Compared with PUF, the water retention of the BBS/PUF biodegradable film improved after BBS modification, which improved the crop growth and increased the crop yield [73].

**Figure 9:**
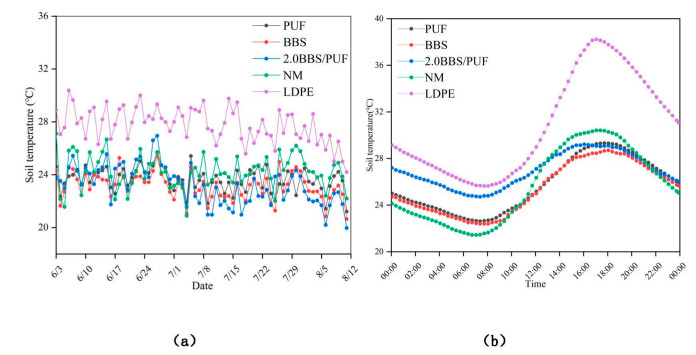
Temperature changes of different treatments, changes in soil temperature during the whole cultivation period (**a**), changes of soil temperature in 24 h, on a typical high-temperature day, 27 June (**b**).

The following is the soil temperature variation curve of the cucumber growth period (Figure 9a) and a typical high-temperature day on June 27 (Figure 9b). The figure shows that the soil temperature of LDPE film mulching during the whole growth period was significantly higher than the other treatments. The average temperatures of PUF, BBS, 2.0BBS/PUF, NM, and LDPE were 23.62 °C, 23.11 °C, 23.19 °C, 24.21 °C, and 27.75 °C, respectively. Compared with the NM and LDPE, the three kinds of biodegradable plastic films could provide suitable soil temperatures for cultivating summer cucumber. On the typical high-temperature day of June 27, the highest temperatures of PUF, BBS, 2.0BBS/PUF, NM, and LDPE were 29.32 °C, 28.69 °C, 29.22 °C, 30.41 °C, and 38.21 °C, respectively, and the temperature differences with the lowest temperature were 6.70 °C, 6.30 °C, 4.51 °C, 8.97 °C, and 12.57 °C, respectively. Compared with NM and LDPE, the three biodegradable mulch films had lower temperature difference changes, and the temperature change range of 2.0BBS/PUF was the smallest, indicating that 2.0BBS/PUF had the highest buffering capacity for the temperature of the cucumber root zone during the high-temperature stage in summer. A low-temperature difference is beneficial not only to plant growth but also to soil microbial activity [74]. In the late stage of cultivation, the soil temperature showed a declining trend; the decrease in environmental temperature and the changing trend between the treatments was mainly the same [75].

## 4. Conclusions

The cellulose and lignin in BBS reacted with PUF resin to improve the viscosity of the modified PUF resin and reduced the content of free formaldehyde. The 2%BBS modified PUF resin (2.0BBS/PUF) accelerated the curing process of the PUF resin, forming a flexible macromolecular network structure with low thermal degradation residues. The biodegradable film prepared with PUF, BBS, and 2.0BBS/PUF as the adhesive had good water retention. Compared with PUF film, the degradation rate of BBS-modified film increased by 17.53%. Meanwhile, 2.0BBS/PUF film had the most buffering capacity for the cucumber root zone temperature and had the lowest temperature difference in the summer high-temperature stage, improving the soil environment and promoting crop growth. This research opens new markets for using lignocellulosic waste biomass as an agricultural mulch, offering a renewable, biodegradable, and environmentally friendly product. However, further research is still needed, to determine the factors for formulating novel ultra-low-formaldehyde-emission UFs with optimal properties and to broaden their application.

## Figures and Tables

**Table 1:** Composition of biosulfonates A and B.

Component	C%	H%	N%	S%	C/N	H/C	C/S
Biosulfonate A	24.88	3.57	0.32	10.51	78.14	1.72	6.31
Biosulfonate B	25.08	4.01	0.51	10.74	49.19	1.92	6.23

**Table 2:** Physical properties of PUF and BBS-modified PUF resins.

Resin	pH	Viscosity (mPa·s)	Free Formaldehyde (%)	Solid Content (%)	Curing Time (s)
PUF	7.2 ± 0.10	297.97 ± 0.68	0.54 ± 0.01	52.95 ± 0.106	65 ± 1
1.0BBS/PUF	7.86 ± 0.08	432.33 ± 2.43	0.41 ± 0.01	54.59 ± 0.212	75 ± 1
1.5BBS/PUF	8.49 ± 0.12	2343.63 ± 3.38	0.35 ± 0.01	53.41 ± 0.179	79 ± 1
2.0BBS/PUF	8.25 ± 0.11	3319.87 ± 13.97	0.27 ± 0.01	53.86 ± 0.111	81 ± 1
2.5BBS/PUF	8.57 ± 0.09	4043.20 ± 38.79	0.26 ± 0.01	53.15 ± 0.171	84 ± 1

## Data Availability

Data sharing is not applicable to this article.
